# Annual Address to the Montgomery County Medical Society, Dec. 21, 1868

**Published:** 1869-02

**Authors:** P. B. Cook

**Affiliations:** The Retiring President


### CHICAGO MEDICAL EXAMINER.

N. S. DAVIS, M.D., Editor.

VOL. X. FEBRUARY, 1869. NO. 2.

### Original Contributions.

ARTICLE VI.

### ANNUAL ADDRESS TO THE MONTGOMERY COUNTY MEDICAL SOCIETY, DEC. 21, 1868.

By the Retiring President, P. B. COOK, M.D.

All annual festivities, from their very nature, gather around them a peculiar interest that marks no other. There is something in the rolling suns, the stated revolutions of the seasons, the set times for commemorating certain events, that makes them of special interest. They naturally cause us to retrospect the whole year, with its duties, privileges, comforts, blessings, joys, or perchance its afflictions and sorrows.

To the medical profession this year has been one of more than ordinary interest—developing new diseases and new remedies, or a new application of old remedies. Especially has it been a year of youthful mortality. More children have died, according to medical statistics, than for years. Recoveries from confinements have been uniformly slow. Even strong men, who have been sick at all, have suffered long and severely; and sickness generally this year has been of a graver type than usual, when no epidemic has prevailed.

But the profession has been active, vigilant, aggressive, and progressive, and, as a consequence, has been growing in efficiency, knowledge, numbers, journals, colleges, professors, and all the means of vivifying, animating, energizing, and encouraging one another; and the world has witnessed the fact in the rapidly increasing academies of medicine, and other numerous medical associations. As a medical society, we form but a small part of that great body known by the name of Medical Fraternity. But, though small numerically, it is not said that we should be in any other respect. With the mind untrammeled and open to conviction, the heart susceptible to every high emotion, and responding to every noble impulse of a nature cultivated, refined, ennobled, elevated, why should we not, adopting for our motto "*Excelsior*," rise with the rising host higher and yet higher?—not forgetting those things which are behind, still press forward towards the mark of the prize of our high and holy calling, sanctified and dignified by our Lord himself.

But, in our day and generation, we find in medicine and surgery, as in all other branches of business, trade, or profession, there are two grand divisions: one, the untiring, unflinching, energetic, determinate man, with a principle which he loves and follows; the other is an easy, indifferent mediocre, who cares for nothing but the bread and butter of to-day; his motto is, "Let us eat, drink, and be merry, for to-morrow we die." Their advance is slowly, but surely, backwards, which of course very naturally causes them to turn up occasionally with those of bygone ages—perchance among the old French, Germans, Scandinavians—or possibly the old Greeks and Romans. They have heard of Hippocrates, and think him a kind of medical divinity; and that the men of his time possessed all the wisdom of the world. Celsus, Galen, and a catalogue of their time, are supposed to have been wiser than Virchow, Paget, and scores of the men of our own time, who have pushed science far beyond anything the ancients ever heard, or even dreamed of.

Imagine an old Egyptian dissecting and examining the various cells of the human body with a microscope of thirty thousand diameters. What greater contrast could there be with his real calling, *viz.*, that of embalming the human body intact, and devoting all his time to the art of mummy-making? Such being their legitimate calling, how could they, or any of the ancients, know, according to their religion and laws, what anatomy meant? or physiology, or chemistry, as applied to medicine? Where did they ever exhibit any skill in operative surgery, or venesection even? What knew they of such agents as chloroform and chloric ether, or such operations as vaccination and cutaneous injection, simple as they are? And farther back in the ages, a man was declared unclean, and cast without the camp, for so much as touching a dead body. Of course, under such restrictions and laws, nothing was known of the real science of medicine. All was intrusted to the "*vis medicatrix naturæ*" to heal any and every sort of affection.

But, leaving the dim shadows of the far past, let us for a moment consider that which comes nearer home. The past year has been one of wholesome rivalry in most respects. New theories have been propounded, and old ones exploded. In addition to the rivalry which is at once healthful and invigorating, the genuine physician has had fierce opposition to combat and overcome in the legitimate practice of the healing art. The public mind has been, and still is, undergoing the process of education and reform—in doing which, the educators have had to run the gauntlet of interested and ignorant opponents, to contend with unscrupulous competitors, to break down the walls of prejudice, to silence the lying tongue; in brief, they have had to show themselves, as an Apostle said, "Examples of good works in all things: in doctrine showing uncorruptness, gravity, sincerity, sound speech that cannot be condemned; that they which are of the contrary part may be ashamed, having (if they had spoken truth) no evil thing to say."

As we have said, the motto of the true physician is "*Excelsior*"—higher, deeper, broader views of all that pertains to nature and nature's laws. This constitutes the whole catechism of the medical man; the first question of which is, What is anatomy? and the last of which shall be written, and the books closed, by the dazzling light that shall change the face of old Mother Earth like the refiner's fire, and make it once more the Eden it was before the fall—which was the first great cause—least understood—creating the necessity, and continuing to the end of time, the office of physician.

This being true, great diligence is required, that we may grow in the knowledge of Anatomy, which numbers and places every bone, every muscle, every artery and vein, every duct, follicle, and tissue of the whole human frame. Physiology is necessary, in order to a correct understanding of the uses of all that constitutes anatomy in the human economy when in a state of health. Chemistry must be understood before we can know the constituent principles and component parts of the whole body, when fitly joined together by that which every joint supplieth. We must also thoroughly understand Pathology, general and special, which simply means nature disturbed, deranged, diseased, its principal divisions being Nosology, Etiology, Symptomatology, Therapeutics, which treat respectively of the classification, causation, symptoms, and cure of disease. Under this last head Surgery comes in as a part of Therapeutics, and relates in some way to the cure. Materia Medica also comes in here, under the general term Medicine, and is prophylactic, preserving the health, and therapeutic, restoring the health. If there be any other thing pertaining to man or medicine not included in the foregoing statement, it may be found in the motto of Maryland, "*Crescite et multiplicamini;*" and in that memorable line of Juvenal, "*E cœlo descendit gnothese auton.*" From Heaven the saying descended, "*Know thyself.*" This speaks volumes, when it is all told.

Regular medicine requires of him who would worthily be called *physician*, that he should possess a knowledge of all the foregoing branches; and a full knowledge of these implies also an acquaintance with ancient and modern languages, and a goodly amount of the history and literature which they include. Holding up and defending such a standard, it is not wonderful we should have opponents (in an age fast as this) to what requires so much labor. First of all, that class which opposes learning in general, are opposed to us. All those who propose to cure everything by water and steam. All who say

"*Similia similibus curantur.*" All those who reject minerals, and prefer vegetables exclusively. All those who make electricity master of all affections. Those who dispense with drugs altogether, only requiring faith in a few mysterious digital reports. Those who profess to cure private diseases with marvellous secret remedies. All these, and a catalogue we have not time to mention, if they be medical men at all, are but fragments of that glorious old system whose history began with Paradise lost, and shall grow in interest and brilliancy till Paradise be regained. True as the axiom, "The whole is greater than any of its parts," so true is regular medicine—greater and better than any of its parts, which it includes.

The tacit admission of these fragmentary theories accounts for the multitude of practitioners—so-called (though self-constituted) Doctors, and the comparatively few Physicians who have received their M.D. from State-constituted authority. Any one who has brass enough to appropriate that which does not belong to him, may enter this whole class of irregulars any time he pleases, under the existing laws of this State. But every one who would enter the field of regular medicine, and become acceptable members of our Association, must go through the years of patient toil and mental discipline which the State prescribes for those who honestly and justly receive the title of M.D., which means learned in medicine. This distinction between the two great classes aforementioned, and the Rules, or Code of Ethics, which govern them, is clearly understood among medical men, and the sovereign people of Illinois are more and more inclined to reflection on these things. Happy will they be, when everywhere such legislation as New England, New York, and other States have adopted, shall be inserted in her Code.

Legislation based upon the motto, "*Salus populi suprema lex esto*," "The welfare of the people is the highest law." Regular physicians need no law for their own protection; for blood will show; and so will educated skill. It is only a matter of time anywhere; and the time is not far distant when the people everywhere will demand of physicians what they do of ministers of the Gospel, *viz.*, *Education;* and by education we do not mean the simple pouring-in-and-out process between preceptor and pupil, but such a culture of the mind and heart as may enable us to think and act intelligently upon any proposition or subject that may be presented to us; whether from Europe, Asia, Africa, America, or the far-off Isles of the Sea; whether from the regions of eternal snow or eternal summer; whether civilized or savage, they all have their voice, which reveals the secret of their lives, and may subserve a useful purpose, if known to the medical man.

We are creatures subject to the bias of education. Moral philosophers tell us conscience is just what it is educated to be and perhaps that other saying is also true, "We are creatures of habit:" combining the two statements, we have what constitutes the man, *viz.*, his conscience and his habits; the former for the most part controlling the latter, though not always. As we may be greatly profited by travelling, and associating the history of the different nations together, we have been endowed with a constitution and nature fitted to endure almost all zones and climates; and the means for acquiring knowledge are greatly multiplied, by the Pen, the Press, the swift ships, the swifter cars, and swiftest wires; all which are the servants of man, under the Divine ordering, and are to develop the mind, expand the heart, ennoble the affections, and make man altogether a more symmetrical being.

But, among all these facilities and ways for acquiring knowledge, there is no royal road, any more than there is to Mathematics. Every one must dig for it himself. Whosoever undertakes any other plan, will be like the man addressed by the Principal of a Female Academy, telling him he had better take his daughter home, as "she was wanting capacity to go on." The fond father, not understanding his vernacular, replied, "Then I'll buy her one." Those who are wanting in application and brains, will have to try buying what they desire, or, if possible, effect an appropriation some other way. But this constitutes one phase of humanity, and is therefore worthy our study; for the Poet has said, "The proper study of mankind is man;" and the saying is pregnant with meaning.

"Know ye not," says the inspired writer, "that ye are the temple of God?" Ten years ago we read the following graphic comment upon these words, by a clergyman of New York:—"This temple is longer than the material universe, which it comprehends and shall outlast; ampler than the solid earth which, in the body, it circumnavigates with its sails, binds with its roads, ties with its wires, but which, in the spirit and its more powerful thought, it grasps as a very little thing, and shall finally fling from it, as a boy tosses aside his top; broader and more diversified in its faculties and attributes than the country of our pride, with more teeming territories of thought, deeper and stronger rivers of feeling, more widely contrasted climates of temperament and mood, more remote and diversified sections of interest and affection—the eternal house of God—the many-mansioned residence of his moral, intellectual, and spiritual essence." And if the round skull and orbed brain be what certain material philosophers make it, or have asserted it to be, the residence and representative of the soul—the visible temple of God—surely, the terraqueous globe itself contains no sandy Saharas or putrid seas, no deserts and wildernesses, no polar circles and miasmatic jungles, inaccessible to human feet, which compare in extent with the moral wastes and unoccupied regions of thought, and the tangled, trackless spaces in the vast convolutions of the brain, with its boundless tissues, as it is ordinarily used.

If phrenology be true, which I am far from affirming or believing, then the anatomist could detect with his knife all those portions of the human brain which had been used, and those, also, which had never come into the knowledge of its owner. Were it the style to submit every one to this test, when done with the brain, methinks these heavy advertisers, these blow-hards, sounding their own trumpets, would be found occupying but a very small portion of this temple, and that away down in the *basement story—none* of that class ever mounting, step by step, to the topmost pinnacle of observation, study, and attainment in medical science. But, wherever found, they would, doubtless, have their old hobby-horse close by their side; no prancing steeds, with distended nostrils and eyes all dancing with delight, to make quicker time than e'er before, at any gait, would anywhere be seen. These men are *usually* known as men of one idea, possessing but little, if any, power of combination. An astute canvasser for one of the largest insurance companies in the land expressed the same idea to me recently, and in good faith. "Medicine," said he, "is so well arranged now, that an ordinary man could learn all that is necessary in a year." I replied that I thought an ordinary man could only about learn his ignorance of medicine in a year, with *good instruction.* But thousands, acting upon the same belief of this agent, employ the veriest charlatans, and pay them the most exorbitant rates, contented to reap the most disastrous results; when, if the same pay had been given to the truly scientific, he might have come with healing and joy to the otherwise disconsolate and desolate hearts. Such a course, so extensively pursued, as it has been, by the people, discourages many a youthful aspirant to knowledge and fame from his intended ways, and, virtually, inclines him to the shortest cut to the practice, I will not say to the profession. Hence, the necessity for legislation. I do not mean class legislation, but the establishment of a standard to which all practitioners shall come who wish to be intrusted with human life. Such a standard, the older countries of the world have already established; and, as "Westward the star of empire takes its course," so, starting from the far East, legislation has crept along to Ohio, even, and soon, we presume, Illinois will take a stand in defence of her people against one of the worst impositions practised upon man.

To assume knowledge, rights, honors, privileges which we do not possess, and never intend nor expect to, is to have the unblushing impudence of a donkey and the chicanery of the veriest knave. This, of course, is done only where the people are credulous and unsophisticated in matters pertaining to medicine, and where there is no law to prevent imposition of the kind. Under such circumstances, if the people are swindled out of a few hundreds, or even thousands, of their hardlyearned, rigidly-saved money, they have no redress. The fact that he was not a professor, as he said; that he was not an A.M., as he pretended; that he was not an M.D., as he affirmed; nor a Ph. D., as he asserted, all this cuts no figure in the case. Even if he is proven a scoundrel and a devil, in everything but the extent of his knowledge; if he never matriculated in any medical college, it is all of no avail; it was a bargain, and the subject of his deceit has simply been outwitted, circumvented, and fleeced.

We read, in Gibbon's History of the Decline and Fall of Rome, that it was an ancient custom that allies of the Republic who ascribed their safety or deliverance to the success of the Roman arms, and even the cities of Italy who admired the virtues of their victorious general, adorned the pomp of his triumph by their voluntary gifts of crowns of gold, which, after the ceremony, were consecrated in the temple of Jupiter, to remain a lasting monument of his glory to future ages. Would the Legislature of Illinois be as noble, or any member thereof frame and have enacted a law for the proper shield and defence of her people against all manner of medical ignorance and quackery, and attach thereto a penalty, which no one would wish to have repeated, for a violation thereof, then the sovereign people of the State could well afford to make a similar oblation, crowning such a legislator with more than a golden crown, *viz.:* with their gratitude and thanks, which should remain a monument of his glorious memory in all the ages and generations yet to come. The genuine, honest, laborious, self-sacrificing physician, who, by his knowledge, skill, persevering industry, and kindness, saves the life of his fellow, is entitled to as much honor as a Roman soldier for doing the same thing, though in a different way; and many are the people who know and feel this to be true, as an occasional tribute will show. Illustrative of this fact, we give the following quotation, selected by Prof. E. B. Stevens, Editor *Lancet and Observer*, from Dr. Reid, Editor *Western Christian Advocate:* "'Luke, the Beloved Physician.' Thus the Divine Word characterizes the only individual of this profession, of which it speaks. Next to the minister, *often beyond the minister*, is the doctor a household favorite. If he has been with us amid much pain and peril, a deep and ineradicable gratitude is associated with his name and his benignant appearance. There have been times, perhaps, when, in our helplessness, we regarded him as the only arm strong enough to parry the blow that death was aiming at some object of our affections. We have watched the struggle with varying hopes and intense solicitude; but, when victory turned on the side of the doctor, we could have laid down our fortune at his feet, for the service he had rendered us. The doctor comes to our sick-room, day after day; he heeds the summons at night as cheerfully and promptly as if it were no pain to rise from bed and go out into the dark, damp, cold, cheerless streets, and into the chamber of suffering. No hour is his own. Neither sanctuary, lecture-room, parlor, study, nor dining-room, is free from the imperative call. The darker the night, the more howling the storm, the more likely some hypochondriac will be to fancy that he is just about to die, and the attendant must be summoned. Such is this profession; in it no rest is possible; pain, pestilence, dying, are its constant attendants. This profession is distinguished, too, for its extensive charities. As a body, physicians attend as cheerfully upon the poor as upon the rich. Where it is absolutely certain there can be no remuneration, still they are as constantly watching and prescribing. The tone of this profession is nobly above the sordidness of most other pursuits in life. It bases itself and buries itself in the humanity of its calling. It regards itself as set for the alleviation of human suffering, and the preservation of human life. The noblest manifestation of this is in the principle so universally accepted by the profession, that *there should be no secret medicines*." The Professor adds: "Such is a part of the grateful and appreciative tribute which a noble minister of the Gospel has thought right to bestow on physicians; a tribute, we would fain hope, truthfully applicable to us, as a whole profession."

We had spoken of the medical man under the scriptural figure of a temple, which, according to its original signification, was a very complete thing, and implied, first of all, the perfection of the architect, as exhibited in its plan, development, and completion; and, also, how cunning must be the artisan, to execute his every order in preparing each part, so that, when all were placed together, they should have more the appearance of the divine than the human handiwork. So, as artisans, we are to study the plan of this divinely-constructed temple from top to bottom, from centre to circumference; learn all its cells, its numerous passage-ways, its rooms and lights, its doors, with all their hinges and joints, of what material they are each composed, what telegraphic signals are given when they are injured, what instruments and material are necessary in the repair, and how used, what changes may be required of air, position, or place. All this requires not only a knowledge of the temple itself, but also of its surroundings. 1st. Is the air salubrious, the scenery attractive and beautiful; or is it the reverse? Does the patient need the mountain air, with its excess of oxygen, or the sea-breezes, with an excess of hydrogen? Does he need steady heat, or constant cold? What peculiar customs and institutions would bring about a favorable reaction. A knowledge, if you please, of the porches, verandas, observatories, its circumambient walks, its shrubbery, flower-garden, fruit-yard, and all that adorns and utilizes the whole.

But we must not further proceed in enumerating the requirements of the medical man, lest we might seem tedious in the long detail. We have only to say, let business press as it may, the physician must keep up his stock. Nothing, but the imperative calls of his country, should ever turn him aside from the duties which belong to the profession of his choice. The old Latin adage should ever be placed before him in letters of living light, "*Retrorsum non gradum*," "No steps backwards," but, in the language of that brilliant general, whose victories flash like meteors across the land, "*On to the sea!*" On to the sea of perfection in attainments that minister health to the sick, joy to the sorrowing, and life to the dying.

A word, with regard to the object, workings, etc., of the Montgomery County Medical Society, and we are done.

In common with all beneficent associations, we meet for the purpose of mutual recognition and fellowship; our object being to promote the interest, honor, and usefulness of the profession, by the cultivation of medical science and literature, and the elevation of the standard of professional education. Our order of exercises consists, mainly, in the reading of papers, the reports of standing and special committees, the discussion of questions specially selected for that purpose, and the relation of cases that have fallen under the eye of some members of the fraternity, and are by them considered of special interest to the Society. Occasionally, a case of great interest comes before the body in person, which furnishes fresh occasion for fresh investigation and remark. It ought, also, to extend its jurisdiction to the domain of drugs, to insist upon competent persons handling and dispensing them, and insist, also, that none but pure drugs be exposed for sale in the market. With the striking revelations that have been made, relative to the impurity of drugs, in New York and Cincinnati, this suggestion seems imperatively essential to the success of every practitioner of medicine and surgery. True, such vigilance and circumspection, in addition to our present labors in the practice, require almost Herculean strength and activity; but this we must do, if we would attain that degree of excellence we so much admire in others of our own and different professions; for,

"The lives of all great men remind us
We can make our lives sublime,
And, departing, leave behind us
Footprints on the sands of Time.

"Footprints that perhaps another,
Sailing o'er life's stormy main,
A forlorn and shipwrecked brother,
Seeing, shall take heart again.

"Let us, then, be up and doing,
With a heart for any fate;
Still achieving, still pursuing,
Learn to labor and to wait."

